# Connecting for Care: a protocol for a mixed-method social network analysis to advance knowledge translation in the field of child development and rehabilitation

**DOI:** 10.1186/s43058-022-00372-5

**Published:** 2022-12-01

**Authors:** Stephanie Glegg, Carrie Costello, Symbia Barnaby, Christine Cassidy, Kathryn M. Sibley, Kelly Russell, Shauna Kingsnorth, Lesley Pritchard, Olaf Kraus de Camargo, John Andersen, Samantha Bellefeuille, Andrea Cross, Janet Curran, Kim Hesketh, Jeremy Layco, James Reynolds, Paula Robeson, Sharon Straus, Kristy Wittmeier

**Affiliations:** 1 Department of Occupational Science and Occupational Therapy, Faculty of Medicine, University of British Columbia, T325 – 2211 Wesbrook Mall, Vancouver, British Columbia V6T 2B5 Canada; 2 BC Children’s Hospital Research Institute, Vancouver, British Columbia Canada; 3 Sunny Hill Health Centre at BC Children’s Hospital, Vancouver, British Columbia Canada; 4 Moms Against Racism, Victoria, British Columbia Canada; 5 Children’s Hospital Research Institute of Manitoba, Excellence in Neurodevelopment and Rehabilitation Research in Child Health (ENRRICH) Theme, Winnipeg, Manitoba Canada; 6 School of Nursing, Dalhousie University, Halifax, Nova Scotia Canada; 7 IWK Health, Halifax, Nova Scotia Canada; 8 Community Health Sciences and George and Fay Yee Centre for Healthcare Innovation, University of Manitoba, Winnipeg, Canada; 9 Department of Pediatrics and Child Health, Rady Faculty of Health Sciences, University of Manitoba, Winnipeg, Manitoba Canada; 10 Evidence to Care, Teaching & Learning Institute, Holland Bloorview Kids Rehabilitation Hospital, Toronto, Ontario Canada; 11 Bloorview Research Institute, Toronto, Ontario Canada; 12 Department of Occupational Science and Occupational Therapy, Rehabilitation Sciences Institute, University of Toronto, Toronto, Ontario Canada; 13 Department of Physical Therapy, Faculty of Rehabilitation Medicine, University of Alberta, Edmonton, Alberta Canada; 14 Women and Children’s Health Research Institute, Edmonton, Alberta Canada; 15 CanChild Centre for Childhood Disability Research, McMaster University, Hamilton, Canada; 16 Department of Pediatrics, School of Rehabilitation Science, McMaster University, Hamilton, Canada; 17 Department of Pediatrics, Faculty of Medicine and Dentistry, University of Alberta, Edmonton, Alberta Canada; 18 Alberta Health Services, Edmonton, Alberta Canada; 19 Children’s Hospital of Eastern Ontario Research Institute, Ottawa, Ontario Canada; 20 Children’s Treatment Network, Richmond Hill, Ontario Canada; 21 Faculty of Health Sciences, Queen’s University, Kingston, Ontario Canada; 22 Kids Brain Health Network, Burnaby, British Columbia Canada; 23 Children’s Healthcare Canada, Ottawa, Ontario Canada; 24 Dalla Lana School of Public Health, University of Toronto, Toronto, Ontario Canada; 25 Rehabilitation Centre for Children, Winnipeg, Manitoba Canada

**Keywords:** Knowledge translation, Healthcare, Social network analysis, Child development, Rehabilitation, Mixed methods, Protocol

## Abstract

**Background:**

Connections between individuals and organizations can impact knowledge translation (KT). This finding has led to growing interest in the study of social networks as drivers of KT. Social networks are formed by the patterns of relationships or connections generated through interactions. These connections can be studied using social network analysis (SNA) methodologies. The relatively small yet diverse community in the field of child development and rehabilitation (CD&R) in Canada offers an ideal case study for applying SNA. The purposes of this work are to (1) quantify and map the structure of Canadian CD&R KT networks among four groups: families, health care providers, KT support personnel, and researchers; (2) explore participant perspectives of the network structure and of KT barriers and facilitators within it; and (3) generate recommendations to improve KT capacity within and between groups. Aligning with the principles of integrated KT, we have assembled a national team whose members contribute throughout the research and KT process, with representation from the four participant groups.

**Methods:**

A sequential, explanatory mixed-method study, within the bounds of a national case study in the field of CD&R. Objective 1: A national SNA survey of family members with advocacy/partnership experience, health care providers, KT support personnel, and researchers, paired with an anonymous survey for family member without partnership experience, will gather data to describe the KT networks within and between groups and identify barriers and facilitators of network connections. Objective 2: Purposive sampling from Phase 1 will identify semi-structured interview participants with whom to examine conventional and network-driven KT barriers, facilitators, and mitigating strategies. Objective 3: Intervention mapping and a Delphi process will generate recommendations for network and conventional interventions to strengthen the network and facilitate KT.

**Discussion:**

This study will integrate network and KT theory in mapping the structure of the CD&R KT network, enhance our understanding of conventional and network-focused KT barriers and facilitators, and provide recommendations to strengthen KT networks. Recommendations can be applied and tested within the field of CD&R to improve KT, with the aim of ensuring children achieve the best health outcomes possible through timely access to effective healthcare.

**Supplementary Information:**

The online version contains supplementary material available at 10.1186/s43058-022-00372-5.

Contributions to the literature

- This study leverages network theory and knowledge translation (KT) theory to advance our understanding of the structure and impact of social networks on KT in the field of child development and rehabilitation
- The work addresses gaps in the social network analysis literature by using mixed methods and including multiple intersecting participant groups
- Intervention mapping will link identified conventional and network-based KT barriers and facilitators to a range of potential solutions, including network interventions
- Recommendations to strengthen KT and KT networks in the field of child development and rehabilitation will be generated by an expert panel using Delphi methodology

## Introduction

Social connections and relational processes have long been understood to influence knowledge exchange and the adoption of innovation [1]. Despite this awareness, western-research oriented investigations of KT barriers and processes focus most often on individuals or organizations [2, 3]. Given that relationships between people and across organizations within complex systems can impact practice change [4, 5], growing interest exists in applying methods and theories to study social networks to advance KT science and practice [2, 5].

The patterns of relationships or interactions (referred to here as “ties”) between individuals or organizations represent the relational data that can be used to describe social networks [4]. These ties can be formal or informal, in-person or virtual (e.g., email, phone, social media), and can be studied at different levels (e.g., individual, organizational, provincial). This study will explore formal (e.g., clinical, funded research networks) *and* informal (e.g., sharing with a colleague, Facebook group) ties, which collectively support KT in Canada. These ties and the existing gaps between individuals, regions, and organizations that influence KT can be examined empirically using social network analysis (SNA), an emerging approach in KT science [2].

The field of child development and rehabilitation (CD&R) in Canada presents an excellent case to apply SNA methods. This small but diverse field focuses on children with exceptionalities, which may be described in the health care setting as having developmental, behavioral, or neurological conditions, musculoskeletal diagnoses, and/or physical or primary sensory impairments [6]. Research shows that one in 10 Canadian youth live with a (dis)ability [7, 8] and many work closely with CD&R specialists toward child and family-centered goals [9, 10]. The need for innovative approaches to KT in CD&R has been well recognized [11, 12]. The field has been criticized for prioritizing the “art” of rehabilitation over science, and where research does exist, for being slow to take it up in practice [13, 14]. Systematic reviews in certain areas of the field have shown that 14–26% of clinical interventions used are likely ineffective [9, 10], while effective interventions remain difficult to implement [15, 16]. CD&R researchers and health care providers face common barriers to KT, such as inadequate skills, and competing priorities [12, 15, 17]. The range in children’s abilities, clinical presentations, and developmental pathways adds to difficulties in accessing high-quality research on specific patient populations [12, 15, 18]. Gaps in rehabilitation-specific, theory-informed, explicitly tailored, or evidence-based KT strategies may further limit effective KT in CD&R [19–22]. With an over-reliance in the field on didactic educational KT strategies that have demonstrated minimal effectiveness for clinical behavior change [19], persisting evidence-to-practice gaps mean that many children accessing CD&R services do not receive the best care [9, 10], which can negatively impact their participation in daily activities and their quality of life.

Acknowledging that many families experience systemic barriers to accessing CD&R services is also imperative. Racism, trauma, lack of service availability, persisting difficulties related to funding arrangements, and the privileging of western knowledge within the health system [23, 24] all impede access to safe and relevant CD&R services. Barriers to accessing care must be considered within the context of understanding and supporting KT.

**Fig. 1:**
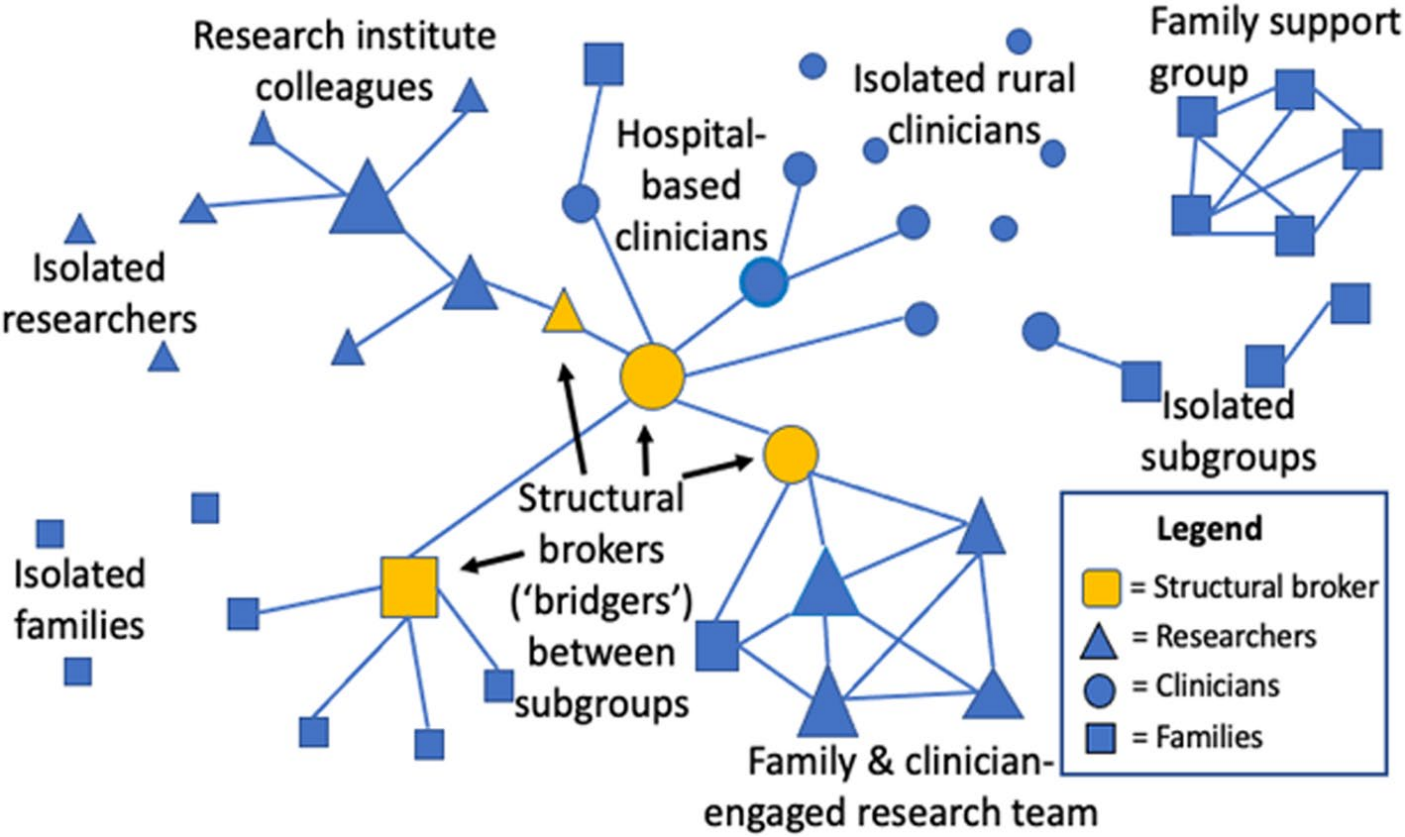
Sample social network graph with nodes (individuals) differentiated by color, size, and shape

SNA can lead to a better understanding of KT barriers and facilitators. The SNA research paradigm is rooted in ethnography and designed to describe and examine social relationships, group dynamics, and information flow [25, 26]. SNA combines social and mathematical theory to quantify relationship attributes, such as tie strength, and the direction of knowledge exchange. SNA software facilitates analysis by quantifying the patterns of ties and gaps and by pictorially illustrating the network (Fig. 1) [27, 28]. These methods present a logical solution to advance the understanding of KT network structure and to direct the design and evaluation of social network-focused KT interventions.

Networks can be measured at the individual (“ego-network”) or “whole network” (e.g., national) level [27]. Relational data are commonly collected using surveys that gather attribute data about the respondent (e.g., age, gender), as well as relational data, by asking “with whom do you connect?” in a particular context [29]. This “name generator” question is accompanied by questions about the named individuals’ characteristics, and the strength of the ties using indicators, such as how long they have been connected, how often they connect, or the level of trust involved [30]. Additional survey questions can contextualize relationships and activities further.

While mixed methods SNA research has been limited [2], pairing SNA surveys with qualitative methods allows for a greater depth of understanding about the network, including the reasons for its structure and *how and why* the KT network functions. Examining the interactions of multiple network member groups (e.g., families, health care providers, KT support personnel, and researchers) allows for within- and between-group analyses to understand the determinants of strong, weak, or absent ties. Once a network’s structure is understood, tailored network-driven interventions can be used to address weak or missing ties (e.g., building on the influence of key individuals, implementing change efforts within specific subgroups, or supporting new interactions) [2, 31, 32]. Despite calls for theory-informed KT interventions that target locally identified barriers and facilitators of change [33], these KT “best practices” are rarely met [22, 34]. The reasons for this gap are diverse, but persistent pitfalls include a mismatch between the problem research aims to “solve” and the strategies used to do so, and a lack of engagement with relevant end-users [34]. To tailor interventions, the barriers and facilitators to KT must be identified, and then explicitly linked (or mapped) to specific KT intervention components [22, 35]. This approach ensures that the intervention employs the hypothesized mechanism(s) of action to effect the desired change [35]. Adding a network lens allows for social/network-driven KT strategies to be considered as interventions within the context of a complex system [5, 36].

The use of SNA in health research and KT has been increasing steadily [2, 37–39]; however, key gaps remain. Glegg et. al highlighted the need to expand SNA research in KT beyond physician groups, to apply mixed methods for a richer understanding of networks, and to incorporate longitudinal designs and deeper analyses to improve clarity about the association between network features and the full spectrum of KT processes and outcomes [2]. Previous research has revealed a tendency toward homophilous ties (e.g., in roles, and gender), highlighting the need for a gender-based approach [40], and for the inclusion of participants representing multiple roles to help identify ways to effectively bridge groups.

SNA can be applied within the field of CD&R to identify strategies to improve knowledge exchange within and across groups, with the goal of improving health outcomes. Formal national research networks, such as the Networks of Centres of Excellence (NCE)-funded Kids Brain Health Network and the Strategy for Patient-Oriented Research-funded CHILD-BRIGHT Network have been created to improve research and KT in CD&R. These networks include KT within their organizational structures. In the clinical sector, CD&R organizations and formal clinical networks have also developed various mechanisms for evidence sharing and uptake [12, 41, 42]. These national, provincial, and organizational groups that include KT within their mandates create a unique opportunity to understand the successes and challenges of existing CD&R KT partnerships and practices and the gaps in linkages that exist. Importantly, SNA research can be used to understand current formal *and* informal social network structures and practices and their intersections, as a baseline measure of connectedness for KT among groups involved in CD&R across Canada. Characterizing the nature of these formal and informal KT ties in CD&R allows for hypothesis generation and testing to improve national KT in this field and to inform KT science more broadly.

## Objectives

The objectives of this study are to:

1. Quantify and visually map the structure of Canadian CD&R KT networks of families, health care providers, KT support personnel, and researchers to identify:
  - Strengths and gaps in the patterns of ties between people and organizations within and across participant groups (i.e., the network structure),
  - Influential individuals and the characteristics that may explain their network positions (e.g., for health care providers, KT support personnel, researchers: profession, experience, gender, geography, ethnicity; for families: gender, geography, ethnicity, child’s age, rarity of diagnosis);
2. Identify family, health care provider, KT support personnel, and researcher perceptions of conventional and network-specific barriers and facilitators of KT within the network and understand the reasons for the CD&R KT network’s structure;
3. Generate recommendations to improve KT capacity within and between participant groups, by linking identified conventional and network-specific barriers and facilitators to KT strategies*.*

## Method

### Study design

A sequential explanatory mixed-method design will be used within a descriptive SNA case study framework [43, 44]. The study will be conducted in three phases to align with each objective of the study, as described in detail below. Definitions and examples of the four participant groups are provided in Table 1. The STROBE Checklist for cross-sectional studies was used to guide reporting (Additional file 1).

**Table 1:** Participant groups, definitions, and examples (not exhaustive)

Participant group	Definition and examples
Family members	Adult family members or caregivers of a child (any age) who have partnered in research or healthcare quality/improvement projects, through individual project level involvement or group involvement such as through a committee, council, or board. Family members who have partnership experience are eligible to complete the “Standard SNA Survey”. Families who have not participated in research or healthcare quality/improvement project partnerships are eligible to complete the anonymous “Family Survey”.
Health care providers	Members of the health care team who provide assessment, consultation, or treatment for children with development or rehabilitation needs. Some examples of healthcare providers are dietitians, Medicine People, nurses, physicians, psychologists, therapists (e.g., physical therapists, occupational therapists, recreation therapists, speech language pathologists), social workers, and traditional healers.
Knowledge translation (KT) support personnel	Elders, implementation specialists, knowledge brokers, Knowledge Keepers, librarians, nurse educators, research coordinators, and others whose formal role includes helping move health-related evidence into action. They may work or volunteer in health care, research, or community organizations.
Researchers	People who design and conduct research as part of their formal role. Researchers can work within universities, research institutes, health organizations, government, or community organizations.

#### Objective 1: Quantify and map the structure of Canadian CD&R KT networks of families, health care providers, KT support personnel, and researchers

##### Data

Online national surveys will be used to quantify the structure of the CD&R KT networks. Data will be collected and managed using REDCap electronic data capture tools, hosted at the University of Manitoba. REDCap is a secure, web-based software platform designed to support data capture for research studies [45, 46]. Intersectionality and KT guidance has been used to inform survey development, administration, and analysis approaches [47]. Survey development and considerations for administration have been informed by conversations with the research team’s family partners, Wisdom Translator, two Knowledge Keepers (one project team member, and a second individual who consulted to our team) and the Children’s Hospital Research Institute of Manitoba Parent Research Advisory Council. A major decision based on these conversations was to create two survey options: a “Standard SNA Survey” and an anonymous “Family Survey”. KT support people, health care providers, researchers, and family members who have participated as partners or team members in research/quality improvement work will be directed to complete the Standard SNA Survey. Family members who have not partnered in research/quality improvement work will be directed to the Family Survey (anonymous, does not include name-generator questions). The decision to include an anonymous Family Survey was made to minimize privacy concerns and to enhance cultural safety within this research.

Both survey options will collect information on participant attributes (typically referred to as participant characteristics, e.g., gender, (dis)ability, ethnicity, CD&R area of interest, years of experience, profession) to understand network composition and to contribute to our understanding of diversity in CD&R related to KT roles. Open-ended questions will be used to collect data on KT network practices and participants’ perceptions of these.

For the Standard SNA Survey, each respondent will be asked a name generator question [38]. This question prompts participants to identify up to five individuals from any of the four participant groups (families, health care providers, KT support personnel, researchers) with whom they connect most closely on KT activities related to CD&R in Canada. To adhere to privacy policies, participants will be notified not to name family members with whom they only interact in a patient-healthcare provider context or participant-researcher context. Thus, family group nominees include only those with professional roles in research and/or health care (e.g., family advisors, patient engagement specialists, family partners or advisors on research projects).

The Family Survey mirrors the Standard SNA Survey in intent, but omits the name generator question (i.e., does not ask for participants to identify/name specific individuals). Instead, participants are asked about the roles of people with whom they connect (e.g., Aboriginal patient liaison, Elder, physician, family member, friend) and the places where they look for information about CD&R (e.g., Friendship Centre, health center, Internet, library). As access to health care services and knowledge sharing about health/health care are intertwined, in both the Standard SNA Survey and Family Survey, questions are posed to explore accessibility of health care, safety/trust in health care, and barriers and facilitators to knowledge sharing.

##### Recruitment

Email, newsletters, blogs, webinars, and social media to leverage professional networks, as well as personal/informal connections, will be used to recruit:

- *Families* through Facebook ads, local and national family support groups, health center Family Advisors, family/patient-engaged research networks, the CanChild/KBHN Family Engagement in Research Training Program through McMaster University, and online groups, such as the Parents Partnering in Research Facebook Group (CanChild/KBHN). We will pursue conversations with community organizations to further support recruitment of families who have not participated in research or health partnerships. Our collaborators (Children’s Healthcare Canada, KBHN, CHILD-BRIGHT, CanChild, Empowered Kids Ontario [EKO]) will support these processes;
- *Health care providers* through provincial regulatory colleges and associations, healthcare/rehabilitation centers, provincial pediatric special interest groups (e.g., EKO), professional councils; and
- *KT support personnel and researchers* through formal provincial and national research networks and collaboratives (e.g., KBHN, CanChild, CHILD-BRIGHT, KT Canada), research institutes, Strategy for Patient-Oriented Research Units, LinkedIn, and relevant university departments.

We have also developed a website (chrim.ca/connectingforcare) that includes information about the study and links to the survey. Social media will be leveraged to direct people to the website for more information. Participants will also be reached through our team’s professional contacts [6].

##### Analysis

For the Standard SNA Survey, relational data (e.g., participants, named contacts, tie strength) will be entered into adjacency matrices, de-identified, and imported into UCINet SNA software [28] to derive network properties. Network visualizations will be generated using NetDraw software [27] (Fig. 1). Attribute data will be analyzed descriptively (e.g., proportions, means/medians). Survey sub-analyses by geographic region will provide insight into specific structural gaps or strengths. QAP regression analysis [48] will be used to explore relationships between network position (dependent variables: degree centrality—i.e., number of ties; betweenness centrality—i.e., bridging position) and individual attributes (e.g., years of experience, education level, gender) for each participant group [25]. To address the known tendency toward homophilous ties (e.g., professional role) within health care networks [40], we will compare heterogenous with homophilous ties to generate hypotheses about conditions that facilitate diverse connections.

For both surveys, analysis of open-ended responses about KT activities will begin with directed content analysis [49]. Data will be coded using a deductive approach using the Theoretical Domains Framework (TDF) qualitative data analysis protocols [50]. The TDF is a theoretical framework that was developed through collaboration between behavioral scientists and implementation researchers [50]. It is based on a synthesis of behavior and behavior change theories that resulted in a framework that categorizes behavioral influences into 14 domains and 84 constructs. The TDF is a validated framework that has been used widely in implementation research across various health care settings [50]. Within our study, the TDF will be used to categorize KT barriers and facilitators into its 14 theoretically derived domains. An inductive coding approach will then be used to generate subcategories of participants’ specific beliefs within the initial TDF coding scheme. A specific belief is a group of similar statements that suggest the belief may influence the target behavior (e.g., knowledge sharing, building KT connections) [50]. Coded data will then be examined to generate themes specific to conventional and network-specific KT barriers and facilitators. Social influence, social capital, and complexity theory [5] (prominent theories applied in SNA [2]) will be combined to supplement the TDF coding framework to guide analysis of the facilitators and barriers related to building KT connections. Coding will be performed by a research assistant/coordinator, and research trainee or member of the research team. All individuals coding data will be trained by a mixed methods researcher and team member (CC) with expertise in using the TDF in qualitative studies, with guidance from a co-PI (SG) on network-specific theory and network properties of interest.

Adhering to the mixed methods paradigm, quantitative data from these Phase 1 surveys will be integrated with qualitative interview data (described below) at multiple points throughout the study to enrich overall data quality and interpretation [26, 43, 51]. For example, quantitative survey data will inform the sampling strategy and interview guide development, and a data triangulation protocol will be used to merge survey and interview data [44, 52, 53].

#### Objective 2: Identify family, health care provider, KT support personnel, and researcher perceptions of conventional and network-specific barriers and facilitators of KT within the network and understand the reasons for the CD&R KT network structure.

##### Data

In Phase 2, consenting participants will take part in a semi-structured qualitative interview. “Intersectionality and KT” guidance for interview administration and analysis will be used to ensure that the safety of participants and that the complexity of lived experience is continuously considered and respected [47, 54]. For those who participated in the Standard SNA Survey, individualized (ego-network) visualizations will be created based on the participant’s survey responses. During the interview, participants will be provided with their individual network map as well as the whole network map, to facilitate discussion of network-specific factors that influence KT, and to elicit potential socially driven strategies to strengthen the network. The whole network map will be provided to participants who completed the Family Survey. An overview of key SNA properties of interest will be provided to help guide the discussion. Probing questions will explore formal networks and informal connections, including identified subgroups in the network of relevance to the participant (e.g., participant group, discipline-based, region-specific).

Interviews will further explore conventional and network-specific KT barriers and facilitators, KT practices, patterns of interaction, and how participants view themselves within the KT network. KT practices may be formal (e.g., webinars, e-newsletters) or informal (e.g., sharing articles, seeking colleagues’ advice, supporting practice change). As previously discussed, issues related to health care access will also be considered in discussions about KT barriers and facilitators. The interview guide will be informed by the TDF with intersectionality enhancements [55], Phase 1 findings, and previous research [6].

##### Recruitment

Stratified purposeful sampling with maximum variation (aiming for 1–2 cases per variation) [53, 56] will guide recruitment. The sampling frame will include individuals who completed the Standard SNA Survey and the Family Survey and provided permission to be contacted for follow-up or future studies. Theoretical sampling will be applied to the Standard SNA Survey respondents, based on quantitative values of network position [53, 56]. Individuals within each participant group with high, typical, and low degree centrality (connectedness), and with high betweenness centrality (indicative of structural brokers who bridge otherwise unconnected subgroups within the network) will be identified. This sample will be refined using purposeful sampling to include 1–2 cases per specified diversity attribute within each participant group, based on intersectionality principles (e.g., geographic region, gender, ethnicity, (dis)ability, profession), as well as representation from subgroups within the network (e.g., formal networks). To enable gender-based analysis, we aim to recruit representative proportions of individuals who self-identify as men and as non-binary from each participant group given their under-representation among health care providers and family partners in CD&R. Sample sizes are scaled relative to a projected smaller population of KT support personnel based on Glegg’s previous research [42], and a larger sample of health care providers to encompass their vast, multidisciplinary population. These principles have yielded sample size estimates of 20 researchers, 24 health care providers, 20 family members, and 12 KT support personnel, for a total of 76 interviews. We will monitor the data as they are collected and analyzed for informational richness and information power, to determine the adequacy of these sample size estimates, and adjust the target number of interviews if indicated [56, 57].

##### Analysis

Audio-recorded interviews will be transcribed for line-by-line directed content analysis [49]. Qualitative data will be coded as per TDF qualitative data analysis protocols as described for the open-ended survey responses in Phase 1 [50]. A triangulation protocol will be used to compare and merge interview data and survey data. This protocol as described below provides a detailed approach to examine meta-themes across findings from different data components that have already been analyzed individually [52]. First, we will create a convergence-coding matrix to display findings from the quantitative and qualitative phases. Next, we will evaluate the findings for convergence, divergence, and discrepancies. This approach focuses on explaining the interconnectedness of the quantitative and qualitative results and provides a deeper understanding of the network’s processes, structures and roles, and their impacts on KT [52]. Responses will be analyzed and reported using relevant structural categories and social processes where sample size allows.

#### Objective 3: Generate recommendations to improve KT capacity within and between participant groups, by linking identified conventional and network-specific barriers and facilitators to KT strategies.

##### Data

Phase 3 will be accomplished through intervention mapping [58] and a Delphi consensus building process [59]. Foundational work by Valente describes four types of network interventions: (1) selecting key actors to influence change; (2) implementing a change initiative within a network subgroup; (3) stimulating peer interaction within the network; and (4) purposely altering the network to bring about change [32]. These interventions have yet to be contextualized explicitly to KT networks. Our approach will delineate the specific socially based KT barriers and facilitators that network interventions can target, in addition to mapping conventional KT interventions to barriers and facilitators. Intervention mapping offers a 6-step framework for designing tailored interventions mapped to defined barriers and facilitators (see Table 2 [58]); we will use the first three steps of the framework in this study, and address the final three in subsequent work.

**Table 2:** The six steps of intervention mapping

1. Describe the problem	
2. Identify desired outcome(s)	
3. Develop the recommendations^a^	
4. Refine the program/intervention	
5. Plan implementation	
6. Plan evaluation	

^a^Modified from “develop the program” [58]

The roots of intervention mapping in community-engaged research underscore the need to align interventions to end-user and context-driven needs [35, 58]. The framework is designed to target multiple levels, allowing us to include individual, organization, system, and network-level barriers and facilitators [5, 36, 58]. Using intervention mapping steps 1 through 3, we will generate recommendations for KT strategies that will be refined through consensus work (described below), and implemented and evaluated in future intervention research. Merged results from the Phase 1 surveys and Phase 2 qualitative interviews will provide us with a description of “the problem” to be addressed (i.e., conventional and network-specific KT barriers in CD&R) for each participant group, the facilitators of KT, and the desired network outcomes (e.g., increased connectivity, leveraging key actors). We will use a combined theoretical and pragmatic approach to intervention mapping [51]. Barriers and facilitators will be mapped to recommended strategies by the study leads (who have also held KT support roles) (KW, SG), 2 parent partners (CC, SB), and 2 health care providers (JA, MN), using the intersectionality-enhanced Behavior Change Wheel (a framework designed to identify ways to promote individual-level behavior change based on TDF barriers/facilitators) [47, 60], Valente’s network interventions (described above) [32], and Dogherty’s taxonomy of facilitation interventions (which itemize change strategies whose mechanisms of action involve social facilitation) [61]. These tools employed within a pragmatic approach will provide a range of interventions that can be applied at the individual or group (network) level. Intersectionality considerations [47] will be included during intervention mapping and expanded upon during the Delphi process (see Table 3).

**Table 3:** Prompts to guide the first three steps of intervention mapping (with a clinician lens applied as an example) [35, 47]

Prompt	Example
1a. Barrier or facilitator	Barrier: Clinical practice location (rural setting, solo practitioner, weak ties to provincial or national networks)
1b. Level(s) of barrier/facilitator (individual, organizational, system, network)	Network (limited individual network and whole network connectedness)
1c. Intersectionality considerations related to barrier or facilitator	Age (young), gender (woman), place of residence (rural)
2. Intended outcome/behavior change	Improved access to knowledge and resources, linkage and frequency of contact with mentor, and # of ties in the network
3a. Intervention component selected to address barrier or facilitator	Modeling, training
3b. How this component can be operationalized	Mentorship relationship with well-connected network member

Once interventions are mapped to their corresponding barriers and facilitators, we will verify the suitability of these pairings through structured engagement with end-users [34]. We will use a consensus process that engages key end-users and KT experts for this aim. The Delphi process is well suited to achieving consensus on complex topics among experts in the field over geographical distances [59, 62, 63], and to compare and integrate data from diverse groups that include patients or families [64, 65]. Using anonymous surveys, Delphi overcomes limitations of in-person consensus building in which a dominant voice or influential individual may sway the group [59, 62]. It also allows participants to respond at their own rate, within a given window, enabling them to process their thoughts before responding [63]. To improve the trustworthiness of the Delphi process, we draw on development and reporting guidance from Hasson [66] and Boulkedid [59].

Prior to the first Delphi round, participants will receive the study objectives and methods, and a summary of the survey and interview findings. They will be presented with the identified KT barriers and facilitators and a logic model that maps each identified barrier and facilitator to one or more intervention recommendation. These intervention pairings with their corresponding barrier/facilitator(s) will form the basis of the Delphi survey. Surveys will be pilot tested by 3–4 individuals from different participant groups prior to finalization and distribution [66]. Data will be collected and managed using REDCap electronic data capture tools, hosted at the University of Manitoba [45, 46].

Delphi surveys are conducted in an iterative manner: In Round 1, the anonymous online survey will list each recommended KT intervention and the barrier(s)/facilitator(s) to which it was mapped. Participants will indicate their level of agreement with the matches using a 9-point Likert Scale [59], with open-text options to suggest alternate intervention components, explain their choices, and request clarification of concepts or matches [67]. Matches receiving a score ≥7 from 75% of participants will be retained as-is. Those deemed not relevant by ≥50% of respondents will be removed [68], or if participants identify acceptable alternatives, these will be considered and revised by research team members for inclusion in subsequent rounds [59]. Qualitative responses will be analyzed using conventional content analysis [49, 66].

In rounds 2 and 3 (if necessary), participants will be provided with quantitative data from the previous round (median, range of scores for each item, and their own scoring) [59], an anonymous summary of qualitative data from round 1, and information to address all participants’ requests for clarification [67]. Round 1 criteria will be used by participants again to indicate their level of agreement with the matches.

##### Recruitment

To enhance participation at each stage of the Delphi process, participants are recommended to have prior interaction with the study team, and be fully informed of the multi-stage process [66]. Participants should be considered experts by training or experience in the area of study [66], and representative of the population for which recommendations are being developed [63]. Interview participants will be informed about the Delphi process in the interview consent form and asked for consent to be contacted for this phase. Consenting participants will be purposively sampled from both surveys for diversity of experiences, geographical representation, and sociocultural factors. Oversampling from the family group will balance academic/clinical expertise with lived experience. The final pool will include 2–3 researchers, 2–3 health care providers, 2–3 KT support personnel, and 4–6 family members. They will join research team members (CC, AC, EH, KH, SK, OK, KMS, SS, LP), 3 parent advisors, and 2–4 additional external KT experts to enhance diversity and expertise. Sample sizes for Delphi surveys range from 5 to >1500 [59, 63]. Akins et al. demonstrated stability of findings with a sample of 23 using bootstrap data expansion methods [63].

##### Analysis

Up to three survey rounds will be conducted using identical methods [59], with equal weighting of responses by participant group, aiming for consensus and response stability [67]. Up to three automated reminder emails per round will be sent to participants using REDCap, to enhance completion rates and validity of results [66, 69]. Up to 8 weeks will be allotted for response, analysis, and preparation of feedback per round [59]. Reporting of the Delphi process will include methodologic reporting, total participant response rate per round, participation rate per participant across rounds, and a full list of final recommendations [59, 66].

Our anticipated output from this objective is a set of recommendations for promising strategies that target conventional (e.g., lack of KT skills) and network-related (e.g., absence of ties, isolated actors) barriers and facilitators to improve capacity for KT within and among participant groups at the provincial or territorial and national levels. These interventions can be tested in future research using a multiple baseline design to monitor network dynamics prior to implementation [70].

## Discussion

To our knowledge, this is the first study to systematically integrate network and KT theory in mapping barriers and facilitators to interventions to enhance KT networks. Results will advance KT science through an in-depth understanding of KT barriers and facilitators from diverse groups, purposefully selected based on network position and considering intersectional categories. The methods we use can be adapted by others to understand KT networks in their own fields. Data from CD&R can then be compared with that from other fields to determine unique and shared determinants of network-based KT. This project also addresses gaps in the SNA literature, through the use of mixed methods and its unique application within CD&R, where only one SNA study has been identified to date [2]. Factors identified as hindering and supporting networks will enable the development of a practical framework to design targeted interventions to enhance conventional and network-based KT processes and outcomes across Canada.

Potential challenges include low recruitment within diversity categories, which would yield incomplete data related to barriers, facilitators, and KT strategies. To mitigate this challenge, we have assembled a pan-Canadian team to facilitate recruitment through its various affiliated institutes and networks. Strong support from national CD&R research networks, such as Kids Brain Health Network, and CHILD-BRIGHT Strategy for Patient Oriented Research Network, will support researcher recruitment. A detailed recruitment plan includes a contact list of all pediatric CD&R organizations and research institutes established for previous research [42] and funds to distribute recruitment material through provincial and national professional associations. Family survey recruitment is co-led by parent partner and regional parent advisors and supported through CanChild/Kids Brain Health Network’s Family Engagement in Research Training Program [71]. This rigorous recruitment strategy will provide the sampling frame to support successful interview recruitment. Our pan-Canadian team will also help to mitigate potential recruitment challenges by supporting local information sharing about the study and its intended outcomes. Remote data collection will limit physical interaction and augment convenience (phone/Zoom interviews, REDCap Delphi surveys) in the context of the ongoing COVID-19 pandemic. Additionally, SNA requires participants to name individuals, which may present ethical concerns or feel culturally unsafe for some. Standard SNA survey data will be anonymized early in the analysis and names will not be included in final reports as per our approved research ethics board-approved study protocols. The Family Survey was developed in response to team members’ concerns about cultural safety. Participants will receive comprehensive explanations of confidentiality safeguards.

Our team recognizes the limitations that we carry as a team of predominantly western-trained researchers and health care providers. We acknowledge that this study was initially conceptualized through a Eurocentric lens and that Indigenous knowledge and Indigenous relationality philosophy [72, 73] are strong systems that predate network and KT theory. We commit to being open to changes in process and protocol that will enhance the safety, relevance, and impact of this work for Indigenous families. Creation of the anonymous Family Survey is one example of how our team does and will respond to concerns raised by our team members or others who raise questions or concerns about the safety and relevance of this work. We are committed to an active decolonization process throughout our study to honor Indigenous knowledges and to learn with and from Indigenous colleagues in this work. Our team’s decolonization journey is as important as every other part of this study, including the interpretation and representation of the information that comes from this work. We have an ethical duty as researchers to be as inclusive as possible, and to create a safe space for all voices and all bodies. As part of our action toward reconciliation, we strive to uphold our duty and responsibility as a research team to welcome and value all the voices at the table as equal partners. We commit to applying the study findings to make sure we are walking alongside all children and families with exceptionalities, toward the best supports and outcomes.

Knowledge translation plans include sharing customized feedback on network structure and KT barriers/facilitators and strategies through discussion with formal research and family networks and represented organizations, as well as any new partners, to facilitate awareness and use of the study’s findings. Our partners have expressed interest in the results to inform the selection and prioritization of KT strategies to support network sustainability as part of their legacy planning. Results will be shared through written reports, academic publications, infographics explaining the network maps, and meetings with these entities’ KT support personnel, executive teams, and general membership as appropriate. We aim to create a venue for continued collaboration between individuals and organizations in Canada with an interest in KT within CD&R. We will document our decolonization journey throughout the study and will share our learnings and any further protocol changes in the spirit of transparency and as part of our KT efforts.

While we have a primary focus on advancing science in the field of KT, this project will also have significant results for CD&R, by characterizing for the first time the national patterns of connections among its multiple participant groups. This insight is important for understanding the dynamics of KT at regional and national levels and offers a baseline assessment of its gaps and strengths. These findings, and the range of perspectives on factors hindering and supporting networks, will enable the development of a practical framework to design targeted interventions to enhance conventional and network-based KT processes and outcomes across Canada. We are excited to test the effectiveness of these network interventions to strengthen KT networks in future research. The integration of a network perspective offers an innovative approach to tailoring KT interventions, through a focus on the socially driven nature of KT, as a process facilitated by and carried out by and between people. These strategies will be employed proactively in partnership with our formal network collaborators to support long-term sustainability of the important gains they have generated within CD&R. Augmenting Canada’s capacity for KT at the local, provincial, and national levels is important to help children achieve the best health outcomes possible through timely access to the most effective healthcare.

We close with words from one of our parent partners: “In my work as a parent in research, and in discussions with other parents, I am keenly aware of the frustration that comes from the time it takes to get good research into practice. I am passionate about this project as a preliminary step in understanding and addressing that gap.”

## Supplementary Information

**Additional file 1 **STROBE Statement—Checklist of items that should be included in reports of *cross-sectional studies*.

## Data Availability

Not applicable.

## Abbreviations

BC: British Columbia
CD&R: Child Development and Rehabilitation
EKO: Empowered Kids Ontario
KBHN: Kids Brain Health Network
KT: Knowledge Translation
NCE: Networks of Centres of Excellence
SNA: Social network analysis
TDF: Theoretical Domains Framework
